# Transcranial low-intensity ultrasound stimulation for treating central nervous system disorders: A promising therapeutic application

**DOI:** 10.3389/fneur.2023.1117188

**Published:** 2023-03-08

**Authors:** Yun-Yun Hu, Gang Yang, Xue-Song Liang, Xuan-Si Ding, De-En Xu, Zhe Li, Quan-Hong Ma, Rui Chen, Yan-Yun Sun

**Affiliations:** ^1^Department of Neurology and Clinical Research Center of Neurological Disease, The Second Affiliated Hospital of Soochow University, Suzhou, China; ^2^Jiangsu Key Laboratory of Neuropsychiatric Diseases, Institute of Neuroscience, Soochow University, Suzhou, Jiangsu, China; ^3^Lab Center, Medical College of Soochow University, Suzhou, China; ^4^Second Clinical College, Dalian Medical University, Dalian, Liaoning, China; ^5^Wuxi No. 2 People's Hospital, Wuxi, Jiangsu, China; ^6^Sleep Medicine Center, Suzhou Guangji Hospital, The Affiliated Guangji Hospital of Soochow University, Suzhou, China

**Keywords:** low-intensity transcranial ultrasound stimulation, neuromodulation, central nervous system diseases, Alzheimer's disease, Parkinson's disease, depression, epilepsy

## Abstract

Transcranial ultrasound stimulation is a neurostimulation technique that has gradually attracted the attention of researchers, especially as a potential therapy for neurological disorders, because of its high spatial resolution, its good penetration depth, and its non-invasiveness. Ultrasound can be categorized as high-intensity and low-intensity based on the intensity of its acoustic wave. High-intensity ultrasound can be used for thermal ablation by taking advantage of its high-energy characteristics. Low-intensity ultrasound, which produces low energy, can be used as a means to regulate the nervous system. The present review describes the current status of research on low-intensity transcranial ultrasound stimulation (LITUS) in the treatment of neurological disorders, such as epilepsy, essential tremor, depression, Parkinson's disease (PD), and Alzheimer's disease (AD). This review summarizes preclinical and clinical studies using LITUS to treat the aforementioned neurological disorders and discusses their underlying mechanisms.

## 1. Introduction

Treatment modalities for central nervous system (CNS) diseases include drug therapy, surgical therapy, and physical therapy. It is a major challenge to deliver drugs to a brain lesion *via* the bloodstream due to the particular anatomical structure of the blood–brain barrier (BBB). Physical treatment modalities offer a novel therapeutic opportunity for neurological disorders, especially for those for which effective drugs are not available. Physical treatment modalities currently available for neurological disorders include transcranial direct current stimulation (tDCS), transcranial magnetic stimulation (TMS), photobiomodulation (PBM), deep brain stimulation (DBS), and low-intensity transcranial ultrasound stimulation (LITUS), among others. Although these treatments show great promise for treating neurological disorders, their use is limited in practical clinical applications due to their invasiveness, their low spatial resolution, and the lack of clear mechanisms. TUS, on the other hand, is non-invasive, boasts high spatial resolution, and has high permeability. This review mainly summarizes the potential application of LITUS in neurological disorders, its underlying mechanisms, and the potential development and challenges in its therapeutic application in the future.

## 2. Current neuromodulation technologies

Before discussing the application of LITUS in the treatment of neurological disorders, we will provide a brief introduction to the other neuromodulation technologies, which are listed in introduction section. tDCS applies low-amplitude direct current through electrodes placed on the scalp, altering cortical excitability and spontaneous neural activity (1–5). tDCS has several advantages, such as cost-effectiveness, convenience, high tolerability, minimal side effects, and easy operability (6). However, the effect of tDCS is not robust enough for some clinical applications due to the characteristics of the electric field and the magnitude of the current produced by electrodes. Its penetration depth also need to be improved. Large individual differences have been observed in the effects of tDCS treatment, even when patients are subjected to the same parameters (7). TMS uses circular or figure-eight coils to produce a rapidly changing magnetic field. Through electromagnetic induction, its magnetic field generates eddy currents that can cause synchronous neuronal activity in targeted cortical areas at a resolution of several centimeters (8–12). On the basis of frequency, TMS can be divided into two categories: low-frequency repetitive TMS (≤1 Hz) and high-frequency repetitive TMS (≥5 Hz) (13, 14). Low-frequency TMS leads to a transient decrease in local cortical activity, while high-frequency TMS increases the excitability of local cortical neurons (13, 14). Additionally, TMS can be sorted into two stimulation modes: intermittent theta burst (iTBS) and continuous theta burst (cTBS). iTBS can cause local cortical excitation, while cTBS temporarily inhibits brain signals (15–17). Although the depth of the target region can be adjusted through coils, the spatial resolution and penetration depth of TMS are limited by the magnetic field conductivity and permeability (18). Researchers have attempted to develop TMS hardware that can specifically affect the human brain, such as the triple halo coil, which modulates excitability in the subcortical brain regions (as deep as 10 cm), and the quadruple butterfly coil, which reduces the volume of stimulation by approximately 70% (19). DBS delivers a continuous flow of current to specific neuroanatomical targets through electrodes that are surgically inserted into the brain (20). The invasiveness of DBS limits its therapeutic application. PBM, on the other hand, is considered a non-thermal technique because it uses non-ionizing radiation in the visible (400–700 nm) and near-infrared (700–1,100 nm) ranges of the electromagnetic spectrum, such as lasers, light-emitting diodes, and/or broadband light, to cause photophysical and photochemical events (21, 22). However, the length and strength of light delivery to the brain always pose challenges (23). Therefore, novel neuromodulation technology that is non-invasive and has a high spatial resolution is required to treat neurological disorders. In this context, LITUS, due to its non-invasive nature and high spatial resolution with millimeter-grade accuracy, has attracted researchers' attention (24–26). A pioneering study by Fry and colleagues in 1958 discovered the neuromodulatory potential of ultrasound stimulation. They discovered that stimulating the lateral geniculate nucleus of the thalamus with ultrasound reversibly inhibited the visual pathway in cats (27). In 2002, after neuroimaging experiments in patients with psychiatric disorders, Bystritsky proposed that ultrasound could be used for neuromodulation with therapeutic benefits for psychiatric and neurological disorders (28, 29). Since then, an increasing number of studies have demonstrated the neuromodulatory effect of ultrasound.

## 3. The characteristics of transcranial focused ultrasound stimulation

Ultrasound is a mechanical pressure wave with a frequency >20 kHz that can penetrate soft tissue at a specific wavelength (30). It has strong penetration, good directionality, and high spatial resolution and is non-invasive (18, 31). With these characteristics, ultrasound is used medically as a diagnostic technique (32). As understanding has deepened, it reveals great potential in the treatment of neurological disorders. Unlike diagnostic ultrasound, which requires a frequency range of 1–15 MHz, therapeutic ultrasound generally uses a specific frequency of approximately 1 MHz (33). Ultrasound is applied clinically using high-intensity or low-intensity acoustic waves (34–36). The peak power levels of high-intensity ultrasound can be >1,000 W/cm^2^, while low-intensity ultrasound is usually 30–500 mW/cm^2^ (37). High-intensity ultrasound has therapeutic effects, which can be achieved by focusing ultrasound on a specific area or point, causing a rapid temperature increase that destroys the tissue. In contrast, low-intensity ultrasound, which produces less energy, inflicts less damage to the tissue. LITUS mostly uses medium-frequency (650 kHz) or low-frequency (220 kHz) ultrasound (38).

With these characteristics, ultrasound is used medically as a diagnostic technique (32). As understanding has deepened, it reveals great potential in the treatment of neurological disorders

**Figure 1:**
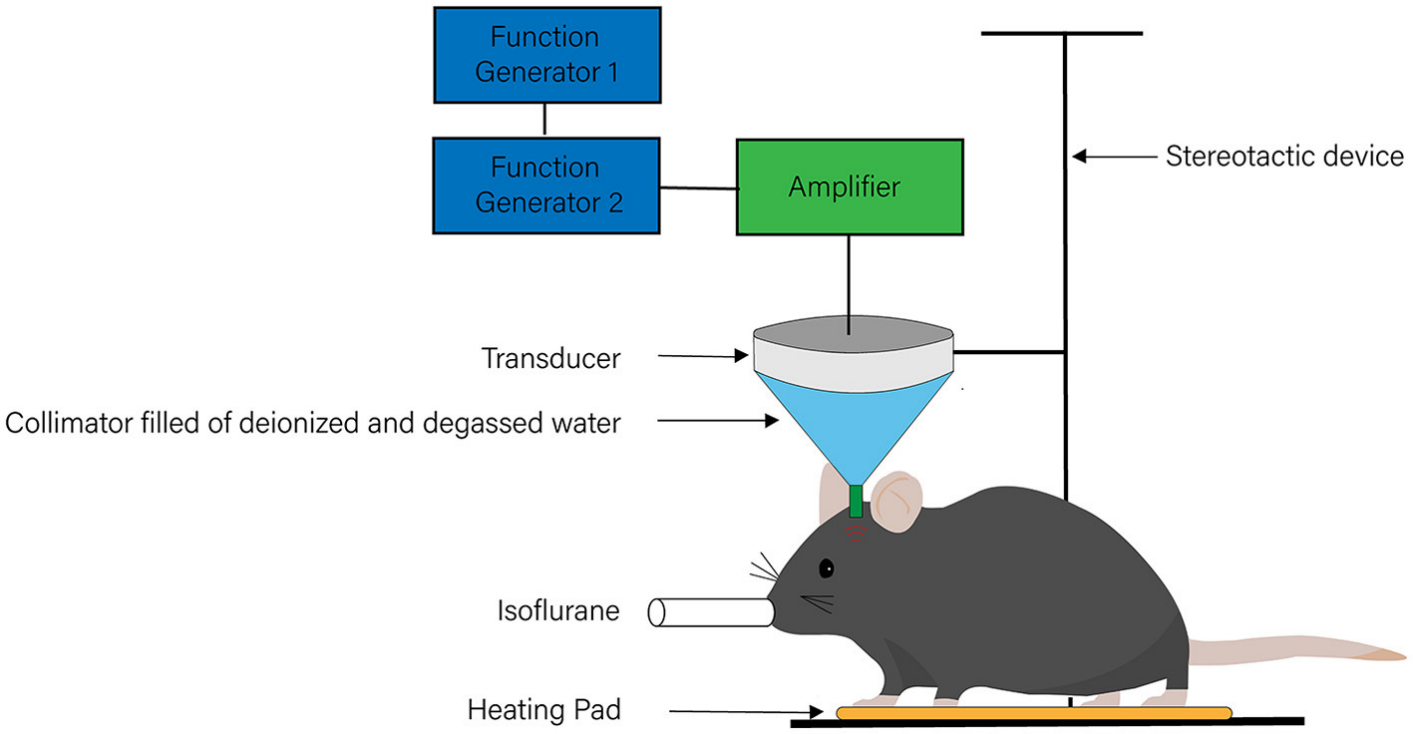
The system of transcranial ultrasound stimulation. The system of transcranial ultrasound stimulation mainly includes a signal generator, a radio-frequency power amplifier, an ultrasonic transducer, a hydrophone, a transducer fixing device, and an ultrasonic coupling agent.

The main components of ultrasonic stimulation systems include a signal generator, a radio-frequency (RF) power amplifier, an ultrasonic transducer, a hydrophone, a transducer fixing device, and an ultrasonic coupling agent (Figure 1). Among these components, the ultrasonic transducer is the core of the whole system, taking advantage of the inverse piezoelectric effect to transform the applied electrical input into mechanical vibration and focus the ultrasound on a target region (18). Curved units that focus the stimulation on oval regions are the most commonly used type of ultrasound transducer (39–46). With this type of ultrasound transducer, the focal volume spans multiple brain subregions in the axial direction of the beam, covering a large area (47) and providing limited target specificity. To overcome this limitation, some researchers use a crossed-beam dual-transducer system (48) to improve the high axial resolution of ultrasound neuromodulation, while others select much higher frequencies, such as 5 MHz, to improve the anatomical specificity (47). The stimulation system enables the adjustment of several key parameters of the ultrasound, including fundamental frequency (FF), pulsed repletion frequency (PRF), stimulation duration (SD), tone-burst duration (TBD), duty cycle (DC), number of tone bursts (NTB), interstimulus interval (ISI), spatial-peak pulse-average intensity (Isppa), and spatial-peak time-average intensity (Ispta) (49, 50). By adjusting these parameters, ultrasound with different frequencies, wavelengths, and acoustic intensities can be generated. Considering its use in the therapy of neurological disorders, it is worth noting that the presence of the skull weakens and distorts the ultrasound signal, affecting the brain tissue (51, 52) and thereby increasing the difficulty of precisely stimulating the brain. Thus, researchers need to set the parameters of ultrasonic stimulation systems with the help of hydrophones to minimize the effects of the skull. Various techniques are available to confirm the neuromodulatory effects of ultrasound stimulation, such as electroencephalography (EEG) (40, 41, 53), electromyography (EMG) (49, 54), functional magnetic resonance imaging (fMRI) (44, 50), and positron emission tomography-computed tomography (PET-CT) (55). In addition, the measurement of extracellular levels of neurotransmitters and metabolic changes can also reflect the effects of ultrasound stimulation (56).

## 4. Ultrasonic stimulation in central nervous system disease

### 4.1. Epilepsy

Epilepsy is a highly prevalent neurological disorder characterized by recurrent episodes of neuronal hyperexcitability or inadequate inhibition (57, 58). It can be caused by brain injury or genetic factors involved in neuronal activity. During seizures, the abnormally synchronous activity in the epileptic foci may spread to other brain regions, eventually causing behavioral abnormalities (59–61). Existing treatments for epilepsy include medical therapy, surgical treatment, and neuromodulation. Medically intractable epilepsy can be treated by removing the epileptogenic focus. However, for some epileptogenic foci that are located in eloquent brain areas or are too numerous, diffuse, or bihemispheric, surgery is not suitable. In such cases, non-invasive neuromodulation offers a viable option for seizure control (62). Laser interstitial thermal therapy (LITT) is a new, minimally invasive technology that has been shown to be effective in treating temporal lobe epilepsy (TLE). It uses a laser through an inserted optical fiber to ablate the epileptogenic focus (63). However, this type of treatment also damages the targeted tissue, causing a decline in brain function and memory, although it is less invasive than surgery (64). Therefore, new technology is indeed needed to reduce the damage and decrease the frequency of seizures. LITUS, a non-invasive physical therapy, has been investigated in preclinical and clinical experiments.

As early as 2011, LITUS was applied to the thalamus of a pentetrazol (PTZ)-induced epilepsy model in rats. After inducing acute seizures in model rats, a series of 0.5-ms-long pulses of sonication were delivered to the thalamic region two times for 3 min, each with a repetition rate of 100 Hz and FF = 690 kHz. EEG recordings revealed that the occurrence of epileptic EEG bursts in rat models was significantly reduced after ultrasound treatment (53). This evidence suggests that LITUS holds promise as a therapeutic tool for the non-invasive suppression of epileptic activity. Some researchers injected kainite (KA) into the CA3 region of the hippocampus of mice to induce mesial temporal lobe epilepsy. In these model mice, ultrasound delayed the onset of status epilepticus (SE) and inhibited acute seizure activity (65). Chu PC and colleagues found that, in the KA-induced epilepsy mouse model, LITUS could reduce the occurrence of seizures, and the effects lasted as long as 7 weeks (66).

Additionally, other research indicates that ultrasound stimulation can decrease the power spectrum intensity of low-frequency (<10 Hz) local field potentials (LFPs), weaken the phase-amplitude coupling intensity between slow and fast nerve oscillations, and increase the time interval of seizures. These results indicate the capability of ultrasound to decrease the power spectrum of LFPs, thereby reducing the onset of epilepsy (67, 68). In addition to exploring the effects of LITUS in rodents, other researchers have attempted to investigate the influence of LITUS in non-human primates. Lin Z and colleagues found that ultrasound stimulation lowered the frequency, duration, and interval of seizures in a penicillin-induced non-human primate model of epilepsy (57). Additionally, Zou's study showed that LITUS decreased the number and duration of seizures in a monkey model of acute epilepsy (69). These lines of evidence strengthen the therapeutic potential of ultrasound stimulation in epilepsy. With the development of this technology, some researchers have begun to apply LITUS to patients with epilepsy.

In a recent study, Lee et al. used LITUS in patients with drug-resistant epilepsy. Two of the patients experienced a decrease in seizure frequency, while one patient showed an increase. The results of LIFUS were only observed in the electrode contacts located at the targeted site, as observed in the SEEG recordings taken before, during, and after treatment. In both patients, LIFUS resulted in a significant reduction in spectral power across all frequency bands. Unfortunately, no correlation was established between these short-term effects and changes in seizure frequency (70). Low-frequency stimulation with magnetic resonance-guided focused ultrasound (MRgFUS) was recently reported to be effective in a patient with medically intractable epilepsy. The patient remained seizure-free for up to 12 months (71). In another study, they developed a device platform to deliver pulsed low-intensity focused ultrasound to the brain region under the hippocampus in humans. After multiple sessions, no adverse events occurred (72). The safety and feasibility of ultrasound stimulation need to be evaluated in future studies with a larger number of participants and a longer duration of follow-up. Thus, to date, the therapeutic evidence of LITUS in epilepsy has mainly been limited to preclinical studies (Table 1), where ultrasound stimulation exhibits great potential in epilepsy therapy. Therefore, more preclinical and clinical studies are still needed to determine how to apply LITUS to the clinical treatment of epilepsy.

**Table 1:** Ultrasound stimulation in epilepsy.

**Refs**.	**Experimental animals**	**Brain targets**	**Protocol of ultrasound stimulation**	**Efficacy**
**Animal Research**				
Min et al. (53)	Male SD rat PTZ-induced acute epilepsy Group 1: PTZ with FUS sonication Group 2: PTZ without FUS sonication Group3: giving FUS sonication without PTZ	The thalamus	FF: 690 kHz TBD: 0.5 ms PRF: 100 Hz Ispta: 100 mW/cm^2^ acoustic focus:3.5 mm in diameter and 6.2 mm in length Deliver twice for 3 min each	(1) The occurrence of epileptic EEG bursts significantly decreased. (2) After FUS, there is less severe epileptic behavior. (3) FUS did not cause any damage to the brain tissue.
Hakimova et al. (65)	Male C57BL/6 mice (5–6 weeks old) KA-induced mesial TLE	Hippocampus	FF: 0.2 MHz PRF: 0.5 kHz TBD:1 ms SD: 30 s 200 acoustic cycles	(1) Ultrasound stimulation reduced the number of seizures in the chronic period of epilepsy. (2) It improved sociability and depressive behaviors in KA model mice.
Li et al. (67)	Male C57BL/6 mice Group 1: KA group Group 2: KA + low-intensity pulsed ultrasound stimulation Group 3: KA + low-intensity continuous ultrasound stimulation	Hippocampus	FF: 500 kHz PRF: 500 Hz DC: 50 % SD: 30 s The acoustic pressure: 0.26 MP The diameter of the hole at the bottom of the conical collimator was 2 mm.	(1) The intensity of the power spectrum in the low frequency (<10 Hz) was significantly decreased. (2) The phase-amplitude coupling strengths between slow and fast neural oscillations were weakened. (3) The interval between seizures was significantly increased.
Lin et al. (57)	Monkeys Penicillin-induced epilepsy model Group 1: penicillin + sham Group 2: penicillin + ultrasound stimulation	The right frontal lobe	FF: 750 kHz PRF: 1 kHz TBD: 300 us SD: 200 ms ISI: 5 s Isppa:2.02 W/cm^2^ The acoustic pressure: 0.35 MPa	(1) Ultrasound stimulation reduces epileptiform activities and behavioral seizures. (2) Ultrasound stimulation activates the interneurons to increase the inhibitory synaptic inputs.
**Refs**.	**Patient characteristic**	**Brain targets**	**Protocol of ultrasound stimulation**	**Efficacy**
**Clinical research**				
Abe et al. (71)	A 36-year-old woman with complex partial seizures without automatism	Hippocampus	650 kHz phased array transducer 10–20s long sonication sessions The final temperature of the target reached 48 °C, and the actual delivered energy was 20757 J.	(1)The patient remained almost seizure-free for up to 12 months. (2) Ultrasound stimulation did not cause any damage to the brain tissue.
Brinker et al. (72)	A 26-year-old female with temporal lobe epilepsy	Hippocampus	FF: 548 kHz PRF: 500 Hz SD: 0.5 s ISI: 7 s DC: 36–50% Ispta: 2.25 W/cm^2^ The acoustic pressure: 0.32 MPa	There were no adverse events.

FF, Fundamental frequency; PRF, the pulse repetition frequency; SD, sonication duration; ISI, inter-stimulus interval; TBD, tone-burst duration; DC, duty cycle; Isppa, the spatial peak pulse mean intensity; Ispta: the spatial peak temporal mean intensity.

Several researchers have discovered some potential mechanisms of the therapeutic effect of LITUS on epilepsy. Chen SG et al. showed that LITUS could change the activity of excitatory neurons, activate GABAergic terminals, downregulate S6 phosphorylation, and decrease pAKT expression (73). Lin Z and his colleagues conducted in-depth studies of the potential mechanisms. They found that LITUS can readjust the imbalance of synaptic inputs to inhibit epileptiform discharges and activate interneurons to increase inhibitory synaptic inputs (57). In conclusion, the aforementioned findings suggest that the therapeutic effects of LITUS are associated with the modulation of neuronal activity and the distribution of inhibitory neuronal axons. The effects and potential mechanisms of LITUS in the therapy of epilepsy need more research in preclinical and clinical experiments.

### 4.2. Essential tremor

Essential tremor (ET) is one of the most common movement disorders among adults and is characterized by postural and kinetic tremors (74, 75). The most recognized feature of ET is a kinetic tremor of the arms, the hands, or the fingers occurring during voluntary movements (76, 77). During voluntary movements, it occasionally occurs in the head, the vocal cords, or other body parts (78). The clinical therapy of ET mainly relies on drug therapy. The first-line oral agents include propranolol and primidone. However, nearly half of patients fail to respond to these oral drugs (79, 80). Before the 1990s, surgical intervention was the only option for patients with severe ET who were unresponsive to oral medicines. The main surgical intervention then was thalamic lesioning. With the advent of DBS, this treatment modality was gradually replaced by DBS. The implantation sites of DBS electrodes are usually the ventral intermediate nucleus (ViM) (81–83) and the caudal zona incerta (cZI) (84, 85). DBS at these two sites alleviates the symptoms of patients with ET with long-term effects. However, the implantation of electrodes can result in side effects for some patients, such as limb paresthesia (which usually improves with programming adjustments), dysarthria, disequilibrium, and skin infections/breakdown (79). As a less invasive approach, MRgFUS is gradually applied in patients with ET, where the thalamic ViM nucleus remains the main action target. MRgFUS thalamotomy exhibits therapeutic effects in patients with ET (86–90) and has been approved by the Food and Drug Administration (FDA) for unilateral treatment of ET (62, 89). Thermal ablation of the thalamotomy still causes side effects similar to those of MRgFUS, including dizziness (early), nausea/vomiting (early), headache (early), flushing (early), ataxia (late), and paresthesias (late) (79). Thus, researchers have explored the potential of neuromodulatory non-thermal LITUS for tremor suppression. When applied to the inferior olivary (IO) system of the harmaline-induced mouse model of ET with an intensity of 27.2 W/cm^2^ (Isppa), LITUS significantly reduced the tremor frequency of model mice (91). This study demonstrates the feasibility of the non-thermal effects of LITUS for tremor treatment. However, more studies are required to establish the technical parameters and mechanism of using low-intensity ultrasound for ET therapy.

### 4.3. Depression

Depression is one of the most common psychiatric disorders. While antidepressant drugs combined with psychotherapy have shown noticeable therapeutic effects, some patients fail to respond to such therapeutic treatments and may experience serious adverse reactions. In preclinical studies, ultrasound stimulation exhibits excellent therapeutic effects on depression. Stimulation with LIFUS on either the prefrontal cortex or the ventromedial prefrontal cortex (vmPFC) attenuated the depressive behaviors of depressed model rats, accompanied by enhanced brain-derived neurotrophic factor (BDNF) levels, whose downregulation is closely linked with depression. Notably, LIFUS improved BDNF levels in the hippocampus of normal mice, suggesting a common mechanism of BDNF signaling induced by ultrasound stimulation.

Moreover, LIFUS enhanced the proliferation and neurogenesis of adult hippocampal neural stem cells (92). The latter is also an essential mechanism underlying depression and is the effect of antidepressant drugs (93, 94). Sha-Sha Yi et al. recently found that LIFUS can alleviate the behaviors of lipopolysaccharide-induced depressed mice. Moreover, the lipopolysaccharide-mediated upregulation of inflammatory cytokines was significantly reduced by LIFUS (95). As in other neurological disorders, the therapeutic effects of some drugs and other factors on depression are limited by the intrinsic properties of the BBB. Relying on its capability of temporarily opening the BBB, MRgFUS together with microbubbles (MBs) successfully and accurately delivered glial cell line-derived neurotrophic factor (GDNF) to the brain, alleviating the symptoms of chronically stressed mice (96). However, the therapeutic potential of non-thermal ultrasound stimulation has not yet been expanded in clinical research. In clinical research, few studies have tested the potential therapeutic effects of tFUS in depression *via* thermal ablation of targeted brain areas. Some researchers have recently investigated whether ultrasound may modulate mood. For example, Joseph L. Sanguinetti et al. found that targeting the right ventrolateral prefrontal cortex *via* tFUS elevated the mood of healthy people after approximately 30 min (97).

Similarly, experiments have shown that LIFUS has the potential to improve mood in healthy subjects (29). Reznik SJ and others performed ultrasound processing on the right frontotemporal cortex of patients with depression and found that it could improve their moods (A double-blind pilot study of transcranial ultrasound (TUS) as a five-day intervention: TUS mitigates worry among depressed patients). These studies provide evidence for the use of LIFUS in the treatment of depression, but more experiments are still needed to verify the improvement effect of LIFUS on depression symptoms.

### 4.4. Parkinson's disease

Parkinson's disease (PD) is the second most common neurodegenerative disease, with clinical symptoms mainly characterized by increased muscle tension, resting tremors, postural instability, and reduced action potentials, accompanied by manifestations of non-motor systems such as autonomic dysfunction and olfactory dysfunction (98). The pathological changes in PD are the gradual loss of nigrostriatal dopaminergic neurons (99, 100). At present, PD therapy mainly relies on levodopa and other drugs to supplement dopamine. Deep brain stimulation (DBS) has exhibited therapeutic effects in reducing the motor symptoms of PD and the side effects associated with long-term dopamine replacement drugs. To date, the stimulation areas have mainly focused on the Vim (101) of the thalamus, the subthalamic nucleus (STN) (102), the globus pallidus interna (GPi) (103), and the cuneiform nucleus (104). However, DBS requires the implantation of electrodes in the corresponding brain regions of patients. It is invasive and poses a risk of infection and cerebral hemorrhage. Jeanmonod et al. reported the feasibility of ultrasound in patients with PD for the first time in 2012. They ablated the fibers that join the thalamus with the globus pallidus by ultrasound delivery. Repeated ultrasound stimulation improved the Unified Parkinson's Disease Rating Scale (UPDRS) score by 57.1% (105). The therapeutic effects of ultrasound stimulation were confirmed by Magara et al. in 2014, who damaged the unilateral pallidothalamic tract in patients with PD using MRgFUS. In this study, 3 months after the surgery, the UPDRS score was significantly improved (106). Afterward, an increasing number of research teams applied ultrasonic ablation in patients with PD, especially with tremor-predominant PD (107–111). However, the treatment modality of high-energy ultrasound ablation also carries the risk of causing serious side effects, such as speech disorders and ataxia (112, 113). As a noninvasive stimulation method, the feasibility of LITUS in PD animal models has been confirmed (Table 2). Hui Zhou et al. first confirmed that the use of ultrasound to stimulate the STN and GP improves locomotor behavior in a 1-methyl-4-phenyl-1,2,3,6-tetrahydropyridine (MPTP)-induced PD mouse model (114). Subsequently, other researchers also observed the beneficial effect of LITUS in PD animal models (114–119). With the advent of ultrasound combined with microbubble technology, an increasing number of researchers have used this technology to enhance blood-brain-barrier permeability to achieve local drug release in the treatment of PD (120–122).

**Table 2:** Ultrasound stimulation in Parkinson's disease.

**Refs**.	**Experimental animals**	**Brain targets**	**Protocol of ultrasound stimulation**	**Efficacy**
**Animal Research**				
Zhou et al. (114)	Male C57BL/6J mice MPTP induced PD model	Subthalamic nucleus (STN) The globus pallidus (GP)	FF: 3.8 MHz PRF: 1 kHz SD: 1 s ISI: 4 s DC: 50 % Ispta: 180 mW/cm^2^ The acoustic pressure: 0.1 MPa The full width at half-maximum was 0.8 mm 30 min daily.	(1) Motor behavior was improved. (2) Ultrasound stimulation can protect TH positive neurons in the SNpc against MPTP-induced cell death. (3) Ultrasound stimulation suppresses cell apoptosis by promoting the ratio of Bcl-2/Bax and inhibiting Cyt C release from mitochondria.
Chen et al. (115)	Male C57BL/6 mice (8-week-old) MPTP induced PD model	The substantia nigra (SN)	FF: 1 MHz PRF: 1 kHz DC: 20% 10 min, 5 times every 24 h	(1) LIPUS treatment can attenuate the central neurotoxicity of MPTP in mice, reduce the loss of tyrosine hydroxylase positive neurons in the substantia nigra pars compacta, and decrease the apoptosis in the section of substantia nigra. (2) The movement and balance dysfunctions were improved (3) There was no tissue damage.
Dong et al. (116)	Male, SD rats 6-OHDA induced PD model	The substantia nigra	FF: 500 kHz PRF: 1 kHz SD: 300 ms TBD: 0.5 ms Isppa: 2.6 W/cm^2^ The total stimulation duration was 10 min, with a total of 200 trials	(1) Ultrasound stimulation reduces the damage of 6-OHDA-induced neurotoxicity in hemi-PD rats.
Sung et al. (117)	Female SD rats 6-OHDA induced PD model	The right striatum	FF:1 MHz PRF: 1 Hz SD: 5 min TBD: 50 ms DC: 5 % Ispta: 528 mW/cm^2^ The half-maximum of the pressure amplitude of the focal zone had a diameter and length of 3 and 26 mm. 5 days per week for a period of 6 weeks	(1) The locomotor function was significantly improved. (2) LIPUS has restorative effects against 6-OHDA neurotoxin by promoting GDNF protein levels and attenuating the LCN2 release in the SNpc of the brain, thereby suppressing neurotoxic cytokines such as IL-1β.
Wang et al. (5)	C57BL/6 mice MPTP induced PD model	Subthalamic nucleus	FF: 500 kHz PRF: 1 kHz DC: 5% SD: 50 ms Isppa: 5.1 W/cm^2^ The maximum ultrasound pressure was 0.39 MPa. The interstimulus interval was 1 s, and the total stimulation time was 5 min	TUS can significantly decrease parkinsonian-related activity in the motor cortex of mice administered MPTP.
Yuan et al. (43)	C57BL/6 mice MPTP induced PD mode	Subthalamic nucleus	FF: 500 kHz PRF: 1 kHz SD: 50 ms DC: 5 % Isppa: 5.1 W/cm^2^ Ispta: 0.255 W/cm^2^ The maximum ultrasound pressure was 0.39 MPa. The diameter of the hole at the bottom of the conical collimator is 4 mm. The interstimulus interval was 1 s, and the total stimulation time was 5 min for each stimulus.	(1) Ultrasound stimulation improves behaviors in mice with MPTP-induced PD? (2) The treatment effect gradually improved as the TUS duration increased.
Zhou et al. (114)	Male C57BL/6J mice MPTP induced PD mode	The motor cortex	FF: 800 kHz PRF: 100 Hz SD: 6 s ISI: 10 s DC: 10% Isppa: 760 mW/cm^2^ 40 min per day The full-width-at-half-maximum was 2.2 mm.	(1) Seven consecutive days of LIPUS stimulation of the motor cortex ameliorated parkinsonian motor deficits. (2) There was no brain tissue injury.

With regard to the underlying mechanisms for LITUS in the treatment of PD, LITUS was observed to alter the extracellular concentration levels of dopamine and serotonin (5-hydroxytryptamine, or 5-HT), suggesting a capability of ultrasound stimulation in the regulation of the local release and uptake, or degradation, of these neurotransmitters (53). The drug treatment for PD in clinical practice relies mainly on the use of levodopa to increase the level of dopamine. This study provides ideas for ways in which LITUS could improve the symptoms of PD. Exposure to MPTP causes a syndrome that mimics core neurological symptoms and the relatively selective dopaminergic neurodegeneration of PD (123, 124). Pretreatment with LITUS inhibited MPP^+^-induced neurotoxicity and mitochondrial dysfunction in PC12 cells and N2a cells (124). Consistently stimulating the motor cortex with LITUS enhanced the levels of T-SOD and GSH-PX in the striatum in MPTP-treated mice (114). These results indicate the role of LITUS in attenuating MPTP-induced mitochondrial dysfunction. Recently, Wen-Shin Song and his team discovered that LIFUS could effectively inhibit glial activation and reduce the phosphorylation of nuclear factor-κB p65 in the substantia nigra pars compacta.

Additionally, it helps to maintain normal levels of neurotrophic factors, dopamine transporters, and tight junction proteins in the blood-brain barrier in PD induced by 6-OHDA (125). Moreover, ultrasound stimulation also exhibits the potential to alter cortical excitability. LIFUS reduced parkinsonian-related brain electrical activity in an MPTP-induced mouse model of PD, as shown by the mean power intensity in the beta band in LFPs, as well as the phase-amplitude coupling intensity between the beta and high gamma bands and between the beta and ripple bands (118). Through these multiple mechanisms, LITUS attenuated dopaminergic neurodegeneration and locomotor deficits in various PD animal models. Notably, improving the sensitivity of ultrasound stimulation with the help of mPrestin (N7T, N308S), an engineered auditory-sensing protein, further ameliorated dopaminergic neurodegeneration and the symptoms of PD in MitoPark mice (mice that exhibit several cardinal features of human PD) (126–128). Thus, preclinical studies strengthen the therapeutic effects of LITUS in PD models. However, the longer-term effects of ultrasound stimulation on PD remain to be further investigated.

### 4.5. Alzheimer's disease

Alzheimer's disease (AD) is a common neurodegenerative disease clinically manifested through the progressive loss of cognitive and memory function, which is pathologically characterized by the accumulation of β-amyloid plaques (Aβ) and hyperphosphorylated tau. The main therapy for AD is drug therapy. One of the challenges of drug therapy for AD is the low efficacy of drugs entering the brain due to the hindrance of the BBB. For example, the anti-Aβ antibody, which helps to clear Aβ load in the brain, has limited capability to enter the brain. LITUS exhibits the capability to temporally open the BBB to allow such drugs to enter the brain. The BBB can be temporarily opened, and then integrity is restored after 4–6 h by LITUS in combination with microbubbles (129, 130). With such a capability, LITUS enhances the delivery of anti-Aβ antibodies or other drugs to the targeted brain regions, thus reducing plaque load, alleviating the cleavage of Tau protein, and rescuing the function of neurons (131). In addition to helping to deliver drugs, the transient opening of the BBB by FUS has beneficial effects on AD model mice. With the use of microbubbles, focused ultrasound stimulation of the hippocampus, the cortex, or even the whole brain in different transgenic AD mouse models, such as TgCRND8, 3xTg, and 5xFAD, without the need for additional therapeutic agents, was efficient for Aβ clearance (132–140) (Table 3). In the context of the transient opening of the BBB, it is worth noting that the BBB in the brains of AD model mice was compromised.

**Table 3:** Ultrasound stimulation in Alzheimer's disease.

**Refs**.	**Experimental animals**	**Brain targets**	**Protocol of ultrasound stimulation**	**Efficacy**
**Animal Research**				
Jordão et al. (131)	Male and female TgCRND8 mice (132–137 days)	The right hemisphere	FF: 0.558 MHz PRF: 1 Hz TBD:10 ms SD:120 s	With the help of microbubbles, ultrasound stimulation locally increases the permeability of the blood brain barrier (BBB).
Jordão et al. (132)	Male and female TgCRND8 mice (4 months)	The right cortex	FF: 0.558 MHz PRF: 1 Hz TBD:10 ms SD:120 s	(1) With the help of microbubbles, ultrasound stimulation locally increases the permeability of the blood brain barrier (BBB). (2) Plaque burden is reduced in cortical brain regions targeted with focused ultrasound stimulation. (3) MRIgFUS-dependent BBB opening allows endogenous immunoglobulin to enter the brain.
Burgess et al. (133)	TgCRND8 mice (7 months)	Hippocampus	FF: 1.68 MHz PRF: 1 Hz TBD: 10 ms SD: 120 s	(1) BBB blood-brain barrier was opened. (2) Ultrasound stimulation improved cognition. (3) Ultrasound stimulation reduced plaque load and increased neuronal plasticity.
Shen et al. (134)	Female 3 × Tg-AD mice (8 months)	Hippocampus	FF: 0.996 MHz PRF: 1 Hz TBD: 10 ms SD: 60 s A peak-rarefactional pressure amplitude of 0.64 MPa	(1) Ultrasound stimulation improved cognition. (2) Ultrasound stimulation ameliorated Aβ deposits and mitigated tau pathology in the hippocampus.
Eguchi et al. (135)	Male 5XFAD mice (14–16 weeks)	Whole brain	FF: 1.875 MH PRF: 6.0 kHz TBD: 17 us SD: 20 min Ispta: 90 mW/cm^2^ Isppa: 99 mW/cm^2^ the number of cycles: 32 The width of the ultrasound beam at each depth of brain tissue ranged from 3.6 to 4.0 mm	Ultrasound stimulation ameliorated cognitive impairments associated with improved cerebral blood flow (CBF).
Bobola et al. (136)	Male 5XFAD mice (6 months)	Hippocampus	FF: 2.0 MHz PRF: 40 Hz TBD: 400 ms Isppa: 190 W/cm^2^ SD: 1 h Chronic: 1 h per day for 5days	(1) Acute ultrasound stimulation can increase the number of microglia around Aβ plaque. (2) Acute ultrasound stimulation reduced Aβ plaque burden.
Lee et al. (137)	5XFAD mice	Hemisphere	FF: 715 kHz PRF: 1 Hz DC: 2 % TBD: 20 ms SD: 60 s	(1) Ultrasound stimulation improved cognition. (2) Ultrasound stimulation enhanced solute Aβ clearance from the brain, but not plaques, to cerebrospinal fluid (CSF) space.
Poon et al. (138)	male and female TgCRND8 mice (7 months)	Hippocampus	FF: 1.1 MH PRF: 1 Hz TBD: 10 ms SD: 120 s The situ pressures of 0.4–0.8 MPa once every other week, for a total of 10 weeks	Ultrasound stimulation reduces the size of existing β-amyloid plaques.
Leinenga et al. (139)	Male APP23 mice (median age, 12.8 months)	Whole brain	FF: 1 MHz PRF: 10 Hz DC: 10 % TBD: 6 s Peak rarefactional pressure is 0.7MPa. The focal zone of the array was an ellipse of about 1.5 mm × 1.5 mm × 12 mm	(1) Ultrasound stimulation engages microglia and promotes the internalization of Ab into microglial lysosomes. (2) Ultrasound stimulation reduces Ab and plaque load.
Leinenga et al. (140)	APP23 mice (21–22 months)	Whole brain	FF: 1 MHz PRF: 10 Hz DC: 10 % TBD: 6 s Peak rarefactional pressure is 0.7 MPa. The focal zone of the array was an ellipse of about 1.5 mm × 1.5 mm × 12 mm 4 times for 8 weeks	(1) SUS treatment increases the number of plaque-associated microglia. (2) SUS Treatment does not reduce the total plaque area but reduces the fraction of larger plaques. (3) SUS treatment reduces fibrillar amyloid. (4) There was no tissue damage.
**Clinical research**				
Lipsman et al. (144)	Patients with early to moderate Alzheimer's disease	Presumed non-eloquent cortex in the right frontal lobe, namely the superior frontal gyrus white matter of the dorsolateral prefrontal cortex (DLPFC)	FF: 220 kHz TBD: 2 ms SD: 300 ms DC: 0.74 % ISI: 2.7 s	Open the BBB in human patients
D'Haese et al. (148)	Patients aged between 50 and 85 years with early AD	Hippocampus and EC	FF: 220 kHz SD: 90 s DC: 50–60% sonication using a range of power of 4–11.5 W	(1) FUS BBB opening is feasible and safe (2) induce a reduction in β-amyloid plaque burden
Nicodemus et al. (149)	Alzheimer's disease patients with age from 40 to 95	The mesial temporal lobe	FF: 2 MHz 520 mW/cm^2^ Eight consecutive, weekly, 1-h	62.5% of patients demonstrated clinically significant improvement on at least one cognitive measure
Beisteiner R	Alzheimer's disease patients	Dorsolateral prefrontal cortex	PRF: 1–5 Hz TBD: 3 us Ispta: 0.1 W/cm^2^ Maximum number of pulses per treatment: 6,000 Maximum peak pressure 25 MPa Every ROI was stimulated twice per session and most patients were stimulated for 4 weeks	(1) No major side effects (2) Neuropsychological scores improve significantly after TPS treatment and improvement lasts up to 3 months and correlates with an upregulation of the memory network (fMRI data)

One explanation for the reduction in Aβ load by LITUS is that it may increase the production of endogenous Aβ antibodies, as Jessica F Jordão et al. found endogenous antibodies bound to Aβ plaques in the cortex of an ultrasound-treated TgCRND8 mouse model of AD (132). Another possibility is that LITUS enhanced the capability of phagocytosis of Aβ by microglia (132, 135, 136, 139). However, in terms of microglial activation, the effects of LITUS seem to be controversial. Eguchiet et al. found that ultrasound stimulation reduced microglial activation in the 5 × FAD transgenic mouse model (135). Leinenga and Gotz et al. observed no change in inflammatory markers in the brains of aged APP23 mice after ultrasound stimulation (139). Thus, the questions of how and whether LITUS-affected glial function contributes to AD pathogenesis remain to be further investigated. In addition, LITUS may enhance neuronal function in AD brains as well. LITUS enhanced axonal neurofilaments in 3 × Tg-AD mice (134) and attenuated the loss of neurons in a 5 × FAD-AD mouse model (137). Burgess et al. observed that ultrasound stimulation increased the number of immature neurons, total dendrite length, and dendrite branching in preexisting or mature neurons in TgCRND8 mice (133). In terms of the molecular mechanisms underlying the beneficial roles of LITUS in AD, some studies have found that this treatment can enhance autophagy, which is compromised in the brains of AD and aging (141, 142).

Through these multiple mechanisms, ultrasound stimulation eventually ameliorates cognitive decline in AD in model animals (133, 135, 137, 139). However, although ultrasound effectively reduces Aβ plaque formation in AD animals, this effect may be attenuated with time after stimulation (132, 143). Therefore, the question of how to prolong the long-term effect of ultrasound on AD therapy remains to be further investigated. The potential of LITUS in AD therapy has also been examined in clinical studies. Ultrasound stimulation combined with microbubbles in the right frontal lobe in patients with AD two times with a 1-month interval successfully opened the BBB but failed to alter the Aβ load (144). The ability of ultrasound stimulation to open the BBB in patients with AD needs further confirmation from other researchers (145–147). Consistent with the observation in preclinical studies that ultrasound stimulation successfully reduces Aβ load, a recent clinical trial showed that Aβ plaques in the hippocampus and entorhinal cortex were reduced 1 week after ultrasound stimulation (interval weeks) in patients with early AD (148). Nicodemus et al. and Beisteiner et al. confirmed that ultrasound stimulation in the cortex of patients with AD for 3 months improved cognitive function (149, 150). Stéphane Epelbaum et al. reported that repeated BBB disruption by ultrasound with microbubbles had a non-significant decline in amyloid accumulation after 4 months (151). The ameliorative effect of LIFUS on pathological parameters in patients with AD in these experiments was based on inducing the opening of BBB. However, Hyeonseok Jeong and colleagues evaluated the safety and efficacy of low-intensity tFUS under the threshold for BBB disruption in patients with AD. They found that, in the absence of an open BBB, the measures of memory, executive function, and global cognitive function were mildly improved (152).

To conclude, LITUS can reduce seizures in models of epileptic disease, improve motor deficits, stimulate dopamine release, reduce EEG activity in PD models, improve depressive phenotypes, and rescue cognitive impairment and neuronal damage in AD models, providing a potential future treatment modality for patients with clear foci who do not wish to undergo invasive treatment.

## 5. Mechanisms underlying ultrasound stimulation-induced neuromodulation

As a mechanical wave, ultrasound can propagate in solids and liquids and exert biological effects on cells and tissues, mainly including thermal effects, mechanical effects, cavitation effects, and so on Table 4. Focusing ultrasound on the ventrolateral nucleus of the thalamus in rats reversibly inhibits somatosensory evoked potentials (SSEPs) spatially in an intensity-dependent manner. The inhibitory effect is consistent in time with the temperature change *in vivo* without producing pathological changes at the tissue level. Stereotactic delivery of thermal energy through optical fibers at the same site also produces similar thermal effects and inhibitory effects (153), suggesting that focused ultrasound may cause neuroinhibitory effects through the thermal effect of ultrasound. Although low-intensity ultrasound does not produce thermal ablation of tissue, the accumulation of ultrasonic energy still increases the local temperature without causing damage. However, the existing evidence is still insufficient to determine whether the increased local temperature caused by ultrasonic focusing is involved in its regulatory mechanism. Tyler et al. (37) applied low-intensity and low-frequency ultrasound to hippocampal slices and mouse brains, and they found that low-intensity ultrasound enhances the electrical activity of neurons by activating voltage-gated sodium channels and calcium channels, as well as improving synaptic transmission in the CNS. The neurons of *C. elegans* expressing TRP4, a stretch-sensitive cationic mechanotransduction channel, are more sensitive to ultrasound stimulation (154). Oh et al. observed that astrocytes are also cellular targets for low-intensity ultrasound stimulation (155). Low-intensity ultrasound-induced neuromodulation is initiated by the opening of TRPA1 channels, a member of the transient receptor potential (TRP) family, in astrocytes. Ca^2+^ entry *via* TRPA1 causes the release of gliotransmitters, including glutamate, in astrocytes, which activates NMDA receptors in neighboring neurons to cause action potential firing. In addition, the expression of a mechanosensitive channel (MscL) also makes neurons or cells more susceptible to activation by low-intensity ultrasound (156, 157), suggesting that ultrasound may also modulate the nervous system by activating mechanosensitive ion channels on the cell surface through its mechanical effects. As a unique physical phenomenon of ultrasound, the cavitation effect has been largely studied and utilized in the treatment of diseases. When ultrasound propagates in fluid or soft tissue containing microbubbles, it can control the contraction and expansion of bubbles. Based on this cavitation effect of ultrasound, combined with intravenous injection of microbubbles, the blood–brain barrier in the brain can be temporarily opened to achieve drug delivery in specific brain regions to achieve precise treatment of the lesion site. For example, focused ultrasound can rescue choline function by delivering selective TrkA agonists into the brains of AD mouse models (158). Hameroff and colleagues propose that ultrasound stimulation at specific megahertz frequency bands can resonate with microtubules, causing them to vibrate when the ultrasound beam angle aligns with their long axis (29). This vibration could then modulate electrical signals in the brain by affecting synaptic plasticity through the connection between microtubules and actin filaments in dendritic spines (159). An increasing number of researchers have recently noticed the presence of auditory confounds during ultrasonic stimulation in humans and animals, considering that the auditory signaling pathways may confound the direct regulatory effects of ultrasound. To investigate whether hearing has an effect during ultrasound stimulation, Guo et al. showed that transection of the auditory nerves or removal of cochlear fluid eliminates US-induced cortical and subcortical activity (160). They indicate that ultrasound activates the ascending auditory system through a cochlear pathway, which activates other non-auditory regions through cross-model projections. In contrast to this observation, Wang et al. found that ultrasound was still capable of inducing neural activity and motor responses, even in chemically deafened PD model mice, suggesting that ultrasound induces neuromodulation *via* multiple action modes that include both direct and indirect effects (118). According to some research, ramping the stimulation onset and offset over several milliseconds can eliminate auditory activation in mice (161). In a clinical experiment, investigators found that a concurrent audio mask applied at the PRF can also reduce auditory perception (162). Other research has shown that ramping and masking TUS stimulation prevent some participants' perception, while the effect of these two methods is not additive (4). Our understanding of the mechanisms underlying ultrasound-induced neuromodulation is currently limited, and more research is needed to advance our knowledge in this area.

**Table 4:** Mechanistic study of transcranial ultrasound stimulation in the treatment of central nervous system diseases.

**Mechanisms of low-intensity transcranial**	
**ultrasound stimulation**	
**Thermal effect**	
The temperature changes	Darrow et al. (153)
**Mechanical effect**	
Change of the opening of ion channels	
Voltage-gated sodium channels	Tyler et al. (37)
Voltage-gated calcium channels	Tyler et al. (37)
TRP4, a stretch-sensitive cationic mechanotransduction channel	Ibsen et al. (154)
Mechanosensitive channel (MscL)	Ye et al. (156), Qiu et al. (157)
**Cavitation effect**	
Opening the blood–brain barrier	Xhima et al. (158)
**Microtubule resonance**	
resonating with microtubules	Hameroff et al. (29)
**Other factors**	
Involvement of auditory signaling pathways	Guo et al. (160), Wang et al. (96)

## 6. Application and development prospects of ultrasonic stimulation

Due to its high spatial resolution and high penetration rate, transcranial ultrasound stimulation is of great significance for the treatment of CNS disorders. It can induce neuromodulation in deep brain regions non-invasively, making it a valuable tool for therapeutic applications. However, this technique is still new, especially for clinical applications. A number of technical problems and challenges urgently need to be addressed by future research (12, 163, 164). First, although preclinical studies show that ultrasound can act on the deep tissue of the brain in the stereotaxic mode, the ultrasonic devices in the existing studies are assembled by individual research teams, which results in a lack of uniform standards for ultrasonic action parameters. From the selection of the devices and the ultrasonic parameters used by each research team, it can be seen that the FF of the focused ultrasound transducer plays a key role in the focal length and focal size of ultrasound (165–167). Second, the voltage wave of ultrasound is converted by the focused ultrasound transducer in a region called the focal spot. The length and width of the focal spot change with the central frequency of the transducer. The larger the frequency, the smaller the focal spot range and the more accurate the active range. However, due to the thermal effect of ultrasound—, the temperature of the action site increases with increasing central frequency, which—may cause thermal damage to the tissue (168, 169). This poses a challenge when selecting different ultrasonic transducers to achieve accurate positioning of the target region. Finally, LITUS requires an ultrasound to act on brain tissue through the skull. Since the acoustic impedance of the skull is greater than that of air, ultrasound will produce different degrees of attenuation when passing through the skull. Therefore, the ultrasonic intensity and energy reaching the target area will be reduced to different levels. Differences in skull thickness among different animals cause varying degrees of ultrasound attenuation upon passing through the skull, which poses challenges for the clinical application of transcranial ultrasound stimulation (170, 171).

In 2003, Norton proposed a new potential technique to stimulate the brain non-invasively; this technique, known as transcranial magnetoacoustic stimulation (TMAS), makes it possible to use LITUS within a static magnetic field (172, 173). TMAS treatment is based on the application of focused ultrasound to a target area within a static magnetic field. In the ultrasonically excited conductive brain, ionic particles induce transient currents generated by Lorentzian forces in a magnetic field. According to Faraday's law, the proportional relationship between the generated electric field and the velocity of ionic particles makes it possible to manipulate the stimulation effect (172, 174). This gives the TMAS an advantage in stimulating specific deep brain regions of small size. Wang H and colleagues first quantified the amplitude and response latency of cortical motor electromyography (EMG) in mice by TMAS compared to LITUS. They found that TMAS could shorten the response time of nerve activity and increase the neuromodulation effect of LITUS on the motor cortex (175). In recent years, more refined and accurate stimulation needs have been proposed with the development of closed-loop brain stimulation techniques, such as DBS, optogenetics, and TMS (176–178). Compared with open-loop brain stimulation, closed-loop brain stimulation can be stimulated as needed according to the received state signal of the brain (179–181), thereby producing the most effective stimulation effect on the brain while reducing the amount of stimulation (182–184). Yang et al. developed a closed-loop transcranial ultrasound stimulation system (CLTUS) for real-time, non-invasive neuromodulation *in vivo*. The application of CLTUS in a mouse model of temporal lobe epilepsy (TLE) inhibits seizures in real time by detecting epileptic echoes online (185). The ultimate purpose of combining ultrasound with different techniques is to enhance its effectiveness in treating diseases. Further studies are needed in the future to prove the feasibility and effectiveness of these different techniques and finally apply them in clinical practice.

Although transcranial ultrasound stimulation is still a new technique, it has already shown great potential in the treatment of CNS disorders in preclinical studies. Therefore, as an emerging treatment modality, it is believed that the aforementioned problems and challenges will be answered and solved in future studies. In addition, TMAS provides low millimeter-scale spatial resolution even in deep brain regions, with a 10-fold higher focus than TMS due to the use of focused ultrasound.

## 7. Conclusion

This article reviews the preclinical and clinical studies of LITUS in the treatment of neurological disorders and summarizes the possible underlying mechanisms. As a non-invasive neuromodulation approach, LITUS exhibits great potential for the therapy of neurological disorders such as epilepsy, ET, PD, and AD, despite their distinct pathological mechanisms. However, the therapeutic application of LITUS for various neurological disorders is far from well-established. Therefore, further exploration is required to enhance the precision and specificity of stimulation by defining the target region and the stimulation parameters in distinct neurological disorders. Moreover, a better understanding of the mechanism underlying the therapeutic effects of LITUS will help accelerate the clinical application of this technology.

## Author contributions

Y-YH, Q-HM, and Y-YS contributed to the conception and design of the study. Y-YH, GY, and X-SL organized the database. Y-YH wrote the first draft of the manuscript. GY, X-SD, RC, Q-HM, and Y-YS wrote sections of the manuscript. D-EX, ZL, and RC provided further insights and co-authored the final manuscript alongside Y-YS. All authors contributed to the final revision of the manuscript and read and approved the submitted version.
